# Identification of the natural product berberine as an antiviral drug

**DOI:** 10.1186/s13568-020-01088-2

**Published:** 2020-09-08

**Authors:** Jiping Shao, Debin Zeng, Shuhong Tian, Gezhi Liu, Jian Fu

**Affiliations:** 1 Hainan Medical University, Haikou, 571199 Hainan China; 2 Hainan Provincial Anning Hospital, Haikou, 571199 Hainan China

**Keywords:** Berberine, Natural product, Small molecule inhibitor, Antiviral activity, Mechanism

## Abstract

Drugs targeting the fusion process of viral entry into host cells have been approved for clinical use in the treatment of AIDS. There remains a great need to improve the use of existing drugs for HIV therapy. Berberine is traditionally used to treat diarrhea, bacillary dysentery, and gastroenteritis in clinics, here our research shows that berberine is effective in inhibiting HIV-1 entry. Native polyacrylamide gel electrophoresis studies reveal that berberine can directly bind to both N36 and C34 to form a novel N36-berberine-C34 complex and effectively block the six-helix bundle formation between the N-terminal heptad repeat peptide N36 and the C-terminal heptad repeat peptide C34. Circular dichroism experiments show that binding of berberine produces conformational changes that damages the secondary structures of 6-HB. Computer-aided molecular docking studies suggest a hydrogen bond with T-639 and two polar bonds with Q-563 and T-639 are established, involving the oxygen atom and the C=O group of the indole ring. Berberine completely inhibits six HIV-1 clade B isolates and exhibits antiviral activities in a concentration-dependent manner with IC50 values varying from 5.5 to 10.25 µg/ml. This compound-peptide interaction may represent a mechanism of action of antiviral activities of berberine. As a summary, these studies successfully identify compound berberine as a potential candidate drug for HIV-1 treatment. As a summary, antiviral activity of berberine in combination with its use in clinical practice, this medicine can be used as a potential clinically anti-HIV drug.

## Key points

- Berberine induces conformational changes to form a novel NHR-berberine-CHR complex.
- The mechanism of action of berberine is that it binds in the pocket of NHR and CHR of gp41.
- Our studies successfully identify berberine as a promising anti-HIV-1 inhibitor.

## Introduction

Human immunodeficiency virus type 1 (HIV-1) causes acquired immunodeficiency syndrome (AIDS). AIDS is an epidemic that has caused significant morbidity and mortality in most countries and poses a serious public health threat worldwide. Although highly active antiretroviral therapy (HAART) can reduce viral load to undetectable level, it cannot completely eradicate the virus because HIV-1 establishes latent infection. Antiretroviral therapy interruption may lead to a rapid rebound of the viral burden in peripheral blood. AIDS patients typically need to take antiretroviral drugs throughout their lives (Chun et al. 2000), which increases the risk of drug resistance and also develops drug-related toxicities. Therefore, development of small-molecule inhibitors with novel mechanisms of action is highly desirable. The Pfizer compound maraviroc binding to the coreceptor CCR5 and suppressing virus-cell fusion has been approved for clinical use (Dorr et al. 2005; Lieberman-Blum et al. 2008; Asano et al. 2014).

**Fig. 1:**
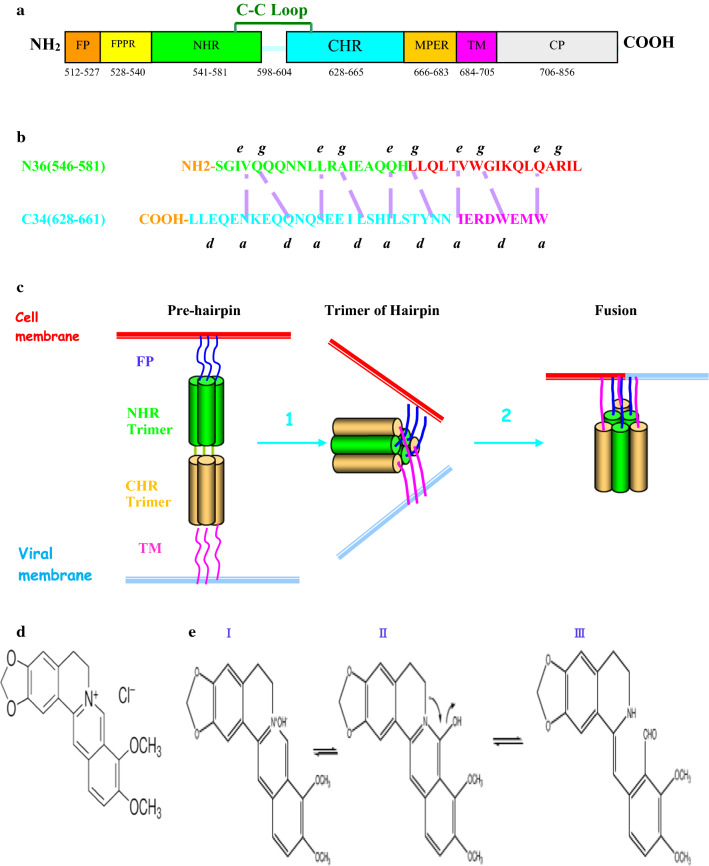
Schematic diagram of HIV-1 gp41. **A** The functional domains of gp41 are indicated, respectively. Residues are numbered corresponding to their positions in HIV-1 HXB2 gp160. **B** The interactions between N36 and C34 of HIV-1 gp41. The purple dashed lines between N36 and C34 indicate the interactions between the residues located at the *e* and *g* positions in the NHR and *a* and *d* positions in the CHR of gp41, respectively. The N36 and C34 sequences are highlighted in green and blue, respectively. The pocket-forming sequence (aa565–581) in N36 and the pocket-binding domain (aa628–635) in C34 are colored in red and pink, respectively. **C** Three steps of HIV-1 entry: pre-hairpin, trimer-of-hairpins, and a stable six-helix bundle core structure. The NHR and CHR helices of gp41 are colored in green and orange, respectively. The 6HB structure is composed of three central NHR helices (green surface) surrounded by three antiparallel CHR helices (orange surface). **D** The chemical structure of berberine. The positively charged nitrogen is indicated. Molecular weight of berberine = 371.8, molecular formula: C20H18ClNO4. **E** The conformational change of berberine. The isomerism qualifies as tautomerism: (I) quaternary-berberine, (II) alcoholic-berberine, (III) aldehydic-berberine

The gp41 subunit of the HIV-1 envelope glycoprotein **(**Env) has emerged as an attractive target for drug intervention. It consists of 345 amino acids (aa) with a molecular mass of 41 kDa shown in Fig. 1A. Gp41 has three major domains: the ectodomain (aa 512 to 683), transmembrane domain (TMD, aa 684 to 705) and the cytoplasmic tail (CT, aa 705 to 856). The ectodomain core structure contains several functional determinants: a fusion peptide (FP, aa 512 to 527); a polar region (PR, aa 525 to 543); the N-terminal heptad repeat (NHR, aa 546 to 581) and the C-terminal heptad repeat (CHR, aa 628 to 661); an immunodominant loop (aa 598 to 604); a Trp-rich region called the membrane-proximal external region (MPER, aa 660 to 683) (Chan et al. 1997). Two peptide fragments denoted N36 and C34 are derived from the N- and C-terminal regions of the ectodomain, respectively (Lu et al. 1995). N36 is a 36-amino acid synthetic peptide that corresponds to the NHR of gp41, C34 is a 34-amino acid synthetic peptide corresponding to the CHR of gp41 (Fig. 1B). Previous studies indicated that N36 and C34 exclusively formed a stable six-stranded helical bundle (6-HB), which brought the viral and cell membranes into close proximity for fusion accompanied by large-scale conformational changes (Fig. 1C) (Weissenhorn et al. 1996, 1997; Munoz-Barroso et al. 1998). The 6-HB core structure has been developed as potential binding sites for developing anti-HIV drugs (Roche et al. 2008). Therefore, inhibiting the formation of 6-HB is an effective way to prevent HIV-1 infection.

Berberine (BBR) is an isoquinoline quaternary alkaloid from medicinal plants, such as Coptis Salisb, Phellodendron Amurense, and Phellodendri Chinensis Cortex, etc. It is commercially available as various salts, such as berberine chloride and hemisulfate. Berberine consists of five rings and two methoxy groups at positions 19 and 20. It has two fluorescent forms in acidic and basic media, which corresponds to a keto-enol tautomerism (Fig. 1D, E). It has a long history of use in Chinese, Ayurvedic, and Southeast Asian traditional medicine. It is used to treat diarrhea, bacillary dysentery, and gastroenteritis. It has also been frequently used for the adjuvant treatment of type 2 diabetes mellitus, ischemic myocardium, hyperlipidemia and hypertension in China (Dong et al. 2013). There is some evidence that berberine has anti-inflammation (Mohan et al. 2017), anti-aging properties (Darzynkiewicz et al. 2014) and induction of apoptosis (Refaat et al. 2015). It has been taken into use against Methicillin-resistant Staphylococcus aureus (MRSA) infections (Stermitz et al. 2000). Most importantly, it has been reported for antiviral activities against numerous viruses (Hayashi et al. 2007; Wu et al. 2011; Varghese et al. 2016; Wang et al. 2017; Yan et al. 2018). Although berberine has been studied extensively, significant questions concerning its binding mode of a small molecule inhibitor of 6HB formation and the mechanism of action remain largely unclear. Here we aim to discover its antiviral mechanism and potential molecular targets and identify it as a promising anti-HIV drug that warrants further investigation.

## Materials and methods

### Materials

HIV-1 plasmids used for the production of pseudotyped virus particles and various cell lines including MT-2 and TZM-bl were obtained from the National Institutes of Health AIDS Research and Reference Reagent Program. HEK 293T and HeLa cells were purchased from the American Type Culture Collection (ATCC). The peptides N36 and C34 were synthesized and purified using analytical high-performance liquid chromatography (HPLC), and the isolates were identified using matrix-assisted laser desorption ionization-time of flight mass spectrometry. Berberine was prepared by dissolving 1 mg powder in 1 ml water to make an aqueous solution (1 mg/ml).

### 6-HB complex

A native PAGE (native polyacrylamide gel electrophoresis, N-PAGE) method was used to confirm the inhibitory activity of berberine on the 6-HB formation as previously described (Liu et al. 2005). Briefly, N36 (100 µM in PBS) was preincubated with various concentrations of berberine (0, 0.05, 0.1 mg/ml) at 37 °C for 30 min and mixed with equal molar C34 (100 µM in PBS) at 37 °C for another 30 min incubation, and then the mixtures were loaded into an 18% precast N-PAGE gels (Invitrogen). Agarose gel electrophoresis was carried out at a constant voltage of 100 V at room temperature. The gels were stained with Coomassie Brilliant Blue (CBB) and imaged using a FluorChem 8800 Imaging System (Alpha Innotech).

### Molecular docking analysis

3D structure of gp41 subunit was retrieved from the PDB database. 3D structure of berberine was built as its hydrochloride. Docking calculations were carried out using DockingServer (Bikadi and Hazai 2009). The molecular geometry of berberine was energy-minimized with the MMFF94 force field (Halgren 1998). Essential hydrogen atoms and solvation parameters were added with the aid of AutoDock tools. Affinity (grid) maps of 20 × 20 × 20 Å grid points and 0.375 Å spacing were generated using the Autogrid program (Morris et al. 1998). Docking simulations were performed using the Lamarckian genetic algorithm (LGA) and the Solis–Wets local search method (Solis and Wets 1981), the most stable complex configurations were considered. All the acyclic dihedral angles in the ligand were allowed to rotate freely. Each docking experiment was derived from two different runs that were set to terminate after a maximum of 250,000 energy evaluations. The population size was set to 150. During the search, a translational step of 0.2 Å, quaternion and torsion steps of 5 were applied.

### CD spectroscopy

Circular dichroism (CD) spectroscopy is a valuable method for protein secondary structure prediction. For example, α-helical proteins are a positive band at 193 nm and negative bands at 222 nm and 208 nm, β-helices have positive bands at 195 nm and negative bands at 218 nm, while disordered proteins exhibit negative bands near 195 nm and low ellipticity above 210 nm. The conformational changes of the individual peptides N36, C34 and their mixtures were determined as previously mentioned (Liu et al. 2005). Briefly, N36 was diluted in ddH_2_O and C34 was diluted in PBS to a final concentration of 10 μM. N36 treated with berberine was incubated with an equimolar concentration of C34 at 37 °C for 30 min, and then the CD spectra of N36, C34 and of equimolar mixtures of N36 and C34 in the presence or absence of berberine (0.05 mg/ml) were observed from 260 to 190 nm at a resolution of 0.5 nm and at a scanning speed of 50 nm/min. The absorption spectra of the prepared solutions were corrected by subtracting the background.

### Cell–cell fusion assay

To examine whether berberine affects Env-mediated membrane fusion, we tested syncytium formation using a microscopic fluorescent dye transfer assay. HeLa cells were co-transfected with the NL4-3 proviral DNA and the GFP construct encoding the green fluorescent protein. Cells were seeded in triplicate in 96-well plates and allowed to attach for 24 h. Then the cells were incubated for 30 min at 37 °C in the presence or absence of berberine. Syncytium formation was measured as the proportion of GFP-positive cells. The percentage inhibition was calculated using the equation given below: % inhibition = [(sample-average negative control)/(average positive control-average negative control)] × 100. 50% inhibitory concentrations (IC50) values were calculated as described before (Liu et al. 2005). Data were presented as the mean ± SD for at least three independent experiments (Pleskoff et al. 1997; Harmon et al. 2010).

### Inhibitory activity of berberine on HIV-1 infection

The inhibitory activity of berberine on HIV-1 infection was determined as previously described. HIV-1 pseudotyped viruses were produced by cotransfecting 293T cells with plasmids encoding the virus backbone and HIV-1 Env DNA using the PolyFect transfection reagent (Qiagen) according to manufacturer’s instruction. Envelope-deficient pNL4-3.luc.E-R-was used for backbone DNA. Pseudotyped viruses were harvested 72 h after transfection by centrifugation and filtration of cell culture through 0.22 μm filters, and stored at − 80 °C until use. To test the inhibitory activity of berberine, viruses were mixed with serial dilutions of berberine or T20 (the first approved anti-gp41 drug) for 30 min at 37 °C, and then the mixtures were added to 2 × 10^4^ HOS-CD4-CCR5 cells grown in 96-well plates. Once the cells were collected and washed 3 times with D-PBS, they were lysed and assayed using the Luciferase Assay System (Promega, USA) according to the manufacturer’s instructions. The potential cytotoxicity of berberine on target cells was measured by using the colorimetric XTT assay (Kilby et al. 1998; Chan and Kim 1998).

### Data analysis and statistics

One way ANOVA was performed, followed by pairwise Tukey test (GraphPad Prism 5.0 software). A p-value ≤ 0.05 was considered to be statistically significant. Dose–response curves were created by nonlinear regression model. IC50 values (50% inhibitory concentration) were calculated by fitting a four-parameter nonlinear regression model using statistical software.

## Results

### The effect of different concentrations of berberine on the 6-HB formation

**Fig. 2:**
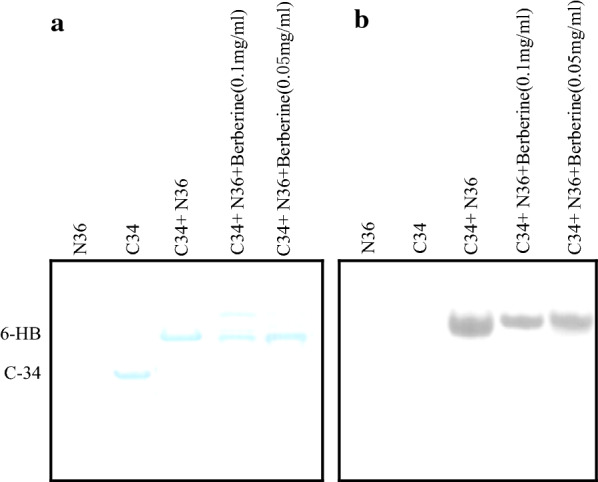
**a** N-PAGE analysis for 6-HB formation by N36 and C34. The preincubated mixtures of the peptide N36 and berberine in phosphate-buffered saline (PBS) at the specified concentrations were added to C34, and then the peptides N36 or/and C34 with or without berberine were loaded into wells formed by combs in the gel**s**. N36 and C34 served as negative controls, C34 + N36 served as a positive control. **b** The 6-HB structure formation by N36 and C34 was measured by Western blot using mAb NC-1

Once berberine blocks the fusion-active six-helix bundle (6-HB) formation by the peptides N36 and C34, it may inhibit HIV-1 Env-mediated membrane fusion and virus entry. To test this assumption, we performed N-PAGE to detect these peptides according to the above protocol. As showed in Fig. 2a, N36 alone exhibited no band (lane1) in the N-PAGE gel since it carried a net positive charge, thus migrating up and off the gel. C34, as a negative control, showed a band in the low position of the gel (lane2). The N36/C34 mixture, as a positive control, displayed a band at the upper side of the gel corresponding to the size of the 6-HB structure (lane3), which is consistent with the previous results (Liu et al. 2005; Jiang et al. 1998). When N36 was preincubated with a high concentration of berberine before the addition of C34, two bands appeared in the gel, the lower one was a 6-HB band with a significant decrease in intensity, the upper one was a novel N36-berberine-C34 complex (lane4). However, if N36 was preincubated with a low concentration of berberine followed by the addition of C34, only a single band was shown in Fig. 2a lane5. This was confirmed by Western blot (Fig. 2b) using a conformation-specific monoclonal antibody NC-1, which specifically detect the 6-HB structure but does not interact with N36 or C34 (Jiang et al. 1998). These results indicated that berberine can bind to N36/C34 and block gp41 6-HB formation, thus inhibiting the fusion between viral and cellular membrane.

### Molecular modeling of compound berberine into the 6-HB structure

**Fig. 3:**
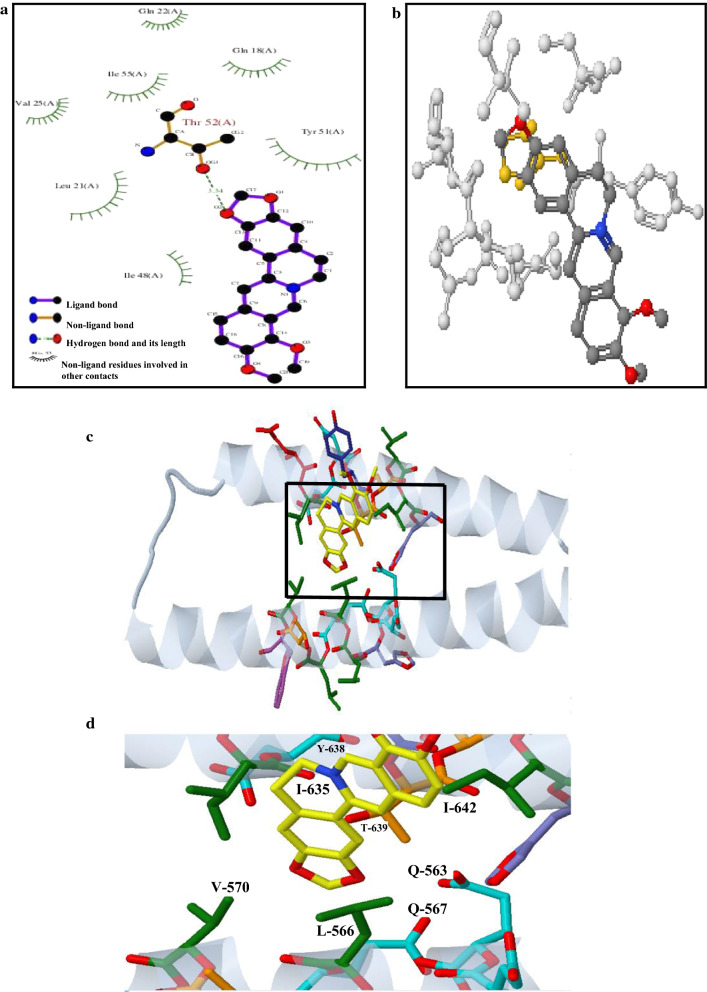
Binding mode and interaction map. **a** Molecular docking of berberine into the 6-HB structure. The location of the potential interacting eight residues were highlighted. Berberine is colored by atom type: Carbon (black), oxygen (red), nitrogen (blue). The hydrogen bond was drawn as green dotted lines. Distance (Å) between interacting atoms was marked in green. **b** 3D representation of different interactions of compound berberine with residues in the binding sites of NHR and CHR of gp41. The highlighted orange spheres represented T-639 and red spheres represented oxygen atom (O). **c** Binding mode of compound berberine in the active region of gp41. The NHR and CHR helices were indicated in silver, the compound was rendered in yellow stick model and the residues were rendered in green, blue and purple sticks, respectively. **d** An enlarged detail view for C. The binding sites of eight amino acid residues were indicated in green, orange and blue, respectively

To discover the binding modes of compound berberine interacting with 6-HB structure, docking simulations of berberine into 6-HB were performed using Molecular Docking Server. Eight side chains had been taken into account in molecular docking strategies by allowing side-chain flexibility. The binding modes of berberine into the cavity binding sites of 6-HB (binding energy: − 8.3 kcal/mol) were presented in Fig. 3 and Table 1. The indole moiety of berberine was pointed toward the mouth of the hydrophobic pocket of gp41 NHR to form p–p interaction and amide-p stacked interaction, and made a π–π stacking interaction with CHR. A hydrogen bond with T-639 and two polar bonds with Q-563 and T-639 were established, involving the oxygen atom and the C=O group of the ring, which appeared to make the most significant contribution to binding. The additional nine hydrophobic interactions with amino acids L-566, V-570, I-635, Y-638, and I-642 were shown in Table 1, Fig. 3c, d, respectively. Moreover, other interactions with Q-563, Q-567, V-570, I-635, Y-638, and T-639 were also involved. The above analysis confirmed that berberine could interact with both the peptides N36 and C34 of gp41 and disrupt the 6-HB formation, this explaining the antiviral activity and mechanism of action of berberine.

**Table 1:** Interaction table

Hydrogen bonds	Polar	Hydrophobic	Other
O2 (*2*) [*3.34*]–THR52 (*OG1*)	O2 (*2*)[*2.96*]–GLN18 (*OE1*)	C11(*16*) [*3.82*]**–**LEU21 (*CG*)	C11 (*16*)[*3.79*]–GLN18 (*NE2, OE1*)
	O1 (1)[3.21]–THR52 (OG1)	C10 (*15*)[*3.65*]–VAL25 (*CG2*)	C13 (*18*)[*3.76*]–GLN18 (*OE1*)
		C12 (*17*)[*3.48*]–VAL25 (*CG2*)	O2 (*2*)[*3.69*]–GLN22 (*CG*)
		C10 (*15*)[*3.12*]– ILE48 (*CB, CG2*)	C17 (*22*)[*3.16*]–GLN22 (*CG*)
		C1 (*6*)[*3.32*]–TYR51 (*CB, CD2, CG*)	O1 (*1*)[*3.12*]–VAL25 (*CB, CG2*)
		C3 (*8*)[*3.70*]–ILE55 (*CD1*)	O1 (*1*)[*3.37*]–ILE48 (*CD1*)
		C7 (*12*)[*3.37*]–ILE55 (*CD1*)	N1 (*5*)[*3.85*]–TYR51 (*CB*)
		C9 (*14*)[*3.87*]–ILE55 (*CD1*)	C10 (*15*)[*3.88*]–THR52 (*OG1*)
		C11 (*16*)[*3.83*]–ILE55 (*CD1*)	C12 (*17*)[*3.21*]–THR52 (*OG1*)
			C13 (*18*)[*3.28*]–THR52 (*OG1*)
			C17 (*22*)[*3.23*]–THR52 (*OG1*)

### Binding of berberine prevented the 6-HB formation

**Fig. 4:**
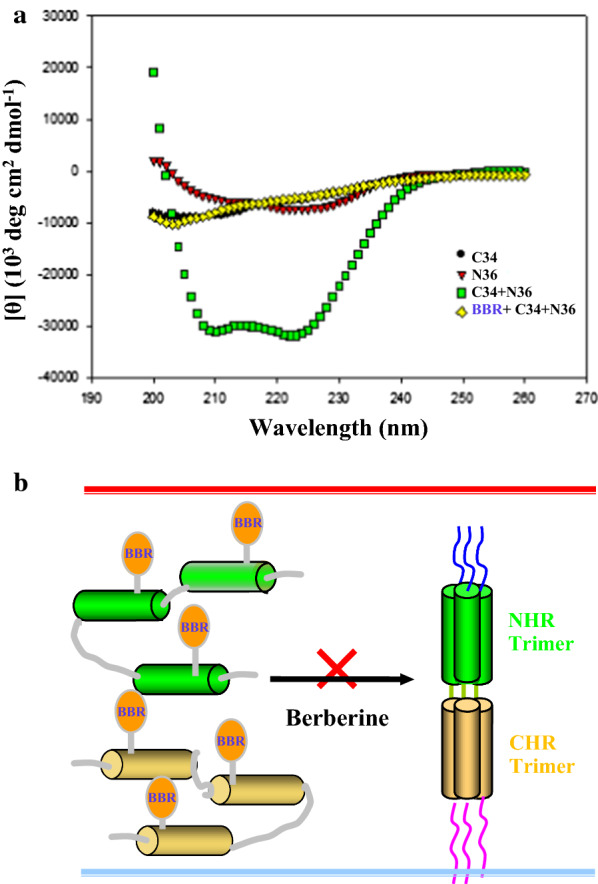
Effect of berberine on the secondary structure of 6-HB structure by CD spectroscopy. **a** The secondary structure of 6-HB in the absence of berberine displayed a typical helical conformation, while lost α-helical conformation in the presence of berberine. N36 and C34 served as negative controls, C34 + N36 served as a positive control. The molar ratio of N36 and C34 in a sample was 1:1. **b** Model of interaction of berberine with NHR and CHR helices of gp41. NHR and CHR helices were indicated in light green and orange, respectively. Berberine was abbreviated to acronyms BBR

To understand the functional effect of the disturbance of natural 6HB structure by berberine, we investigated the impact of berberine on the secondary structure of 6HB. CD spectroscopy is a classic technique to determine the secondary structure composition, such as α-helix, β-sheet and random coil of peptides in the liquid phase, therefore, it has been used for determining the conformational changes of the 6-HB structure formed by the equal molar of N36 and C34. Our CD results indicated that N36 alone in PBS as a control did not exhibit a typical α-helical spectrum, C34 alone as a control showed a random coil spectrum, and the N36/C34 complex in the absence of berberine displayed a typical α-helical secondary structure with a double minimum at 208 and 222 nm, which was a heterotetramer structure with an α-helix with 100% helix content, characterized by the saddle-shaped negative peak in the far UV region of the CD spectrum, however, the CD spectrum of the N36/C34 complex in the presence of berberine was a random coil structure, suggesting that berberine distorted the α-helical structure of the 6-HB (Fig. 4a). These data indicated that berberine might insert into both N36 and C34 of gp41 before the formation of the 6-HB to form a novel N36-berberine-C34 complex (Fig. 2a lane4), and efficiently blocked fusion by causing a local disturbance of the natural 6HB conformation (Fig. 4b), in agreement with previous reports (Liu et al. 2005; Jiang et al. 1998; Miyauchi et al. 2009).

### Berberine inhibited HIV-1 early entry by blocking cell–cell fusion

**Fig. 5:**
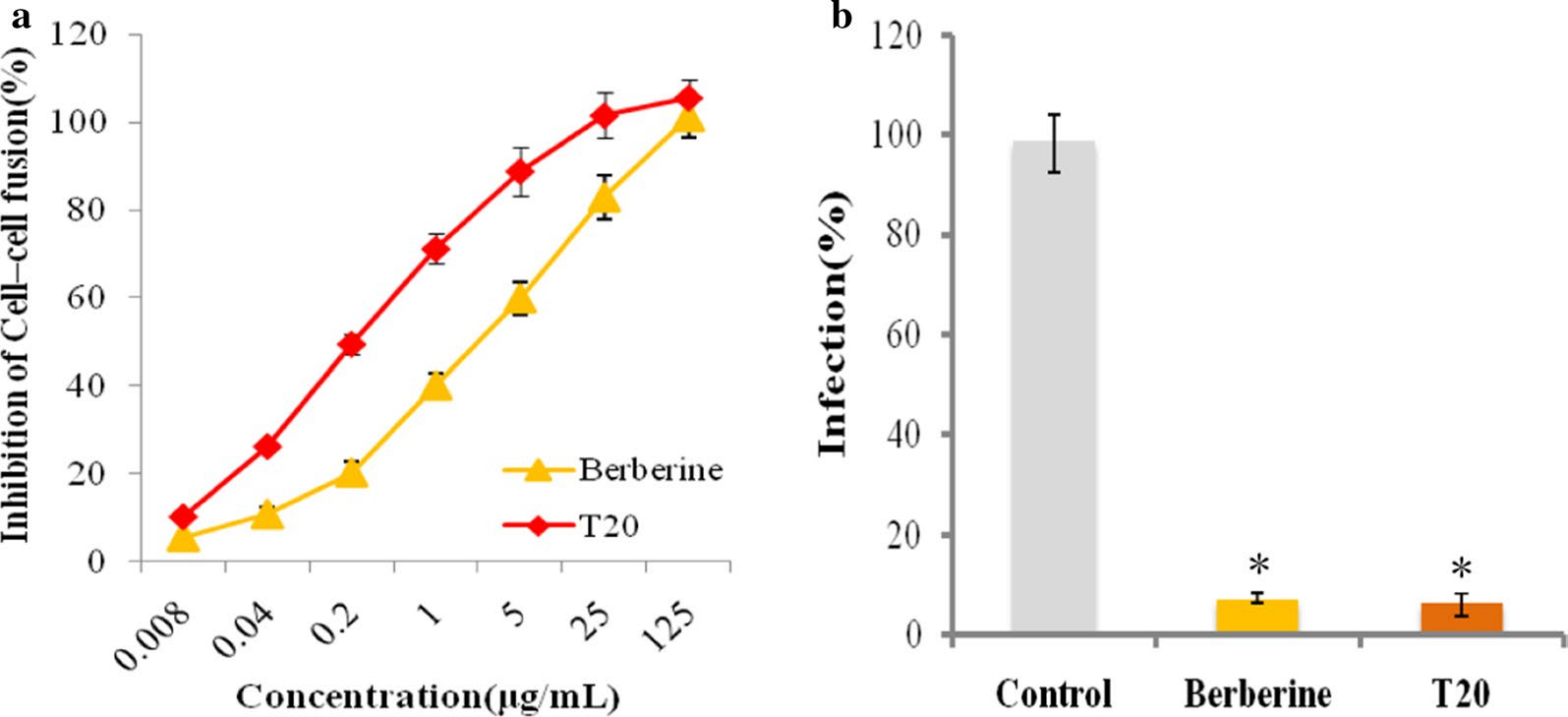
The inhibitory effects of berberine on HIV-1 Env-mediated cell–cell fusion. **a** Inhibition of berberine on HIV-1 Env-mediated syncytium formation. T20 was used as the positive control. **b** Inhibition activity of berberine on HIV-1 infection. Target cells were infected with the HIV-1 virus in the presence or absence of berberine. PBS was used as the negative control

Membrane fusion is an essential step for enveloped viruses’ entry host cells. The formation of syncytia displays a consequence of cell–cell fusion. We next sought to determine the inhibitory activity of berberine on HIV-1 cell–cell fusion. The effector cells were pretreated in the absence or presence of berberine, and then co-cultured with target cells. T20 (DP178, brand name Fuzeon), the FDA approved fusion entry inhibitor, which is a gp41-derived peptide that exhibits HIV-1 entry inhibitory activity, was used as a control. As expected, berberine exhibited potent inhibitory activities on HIV-1 Env-mediated cell–cell fusion, small syncytia were found in the presence of berberine, while large syncytia were observed in the absence of berberine. Moreover, berberine inhibited the syncytia formation in a dose-dependent manner with the IC50 values of 5.773 ± 0.457 μg/ml (Fig. 5a). Berberine impaired viral fusion and showed high inhibition activity compared with the control (Fig. 5b). These results demonstrated that berberine could abolish the function of HIV-1 Env and act as an effective fusion inhibitor of virus-mediated cell–cell fusion. These findings were fully consistent with the above-mentioned results.

### The anti-HIV activity of berberine

**Fig. 6:**
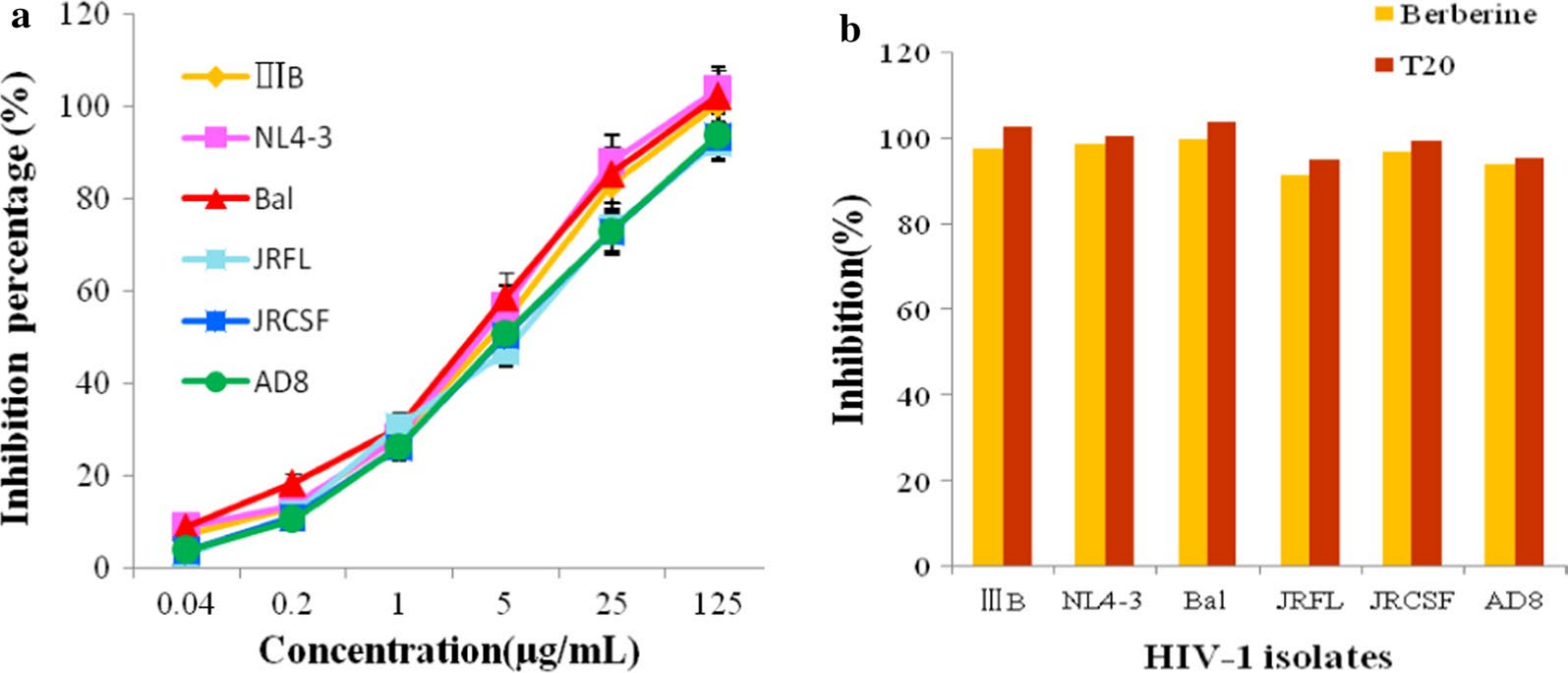
Antiviral activities of berberine. **a** HIV-1 pseudotyped viruses were pre-inoculated with different concentrations of berberine and T20 for 30 min at 37 °C, and then the mixtures were added to target cells grown in each well of 96-well plates. Luminescence was measured and percentage inhibition was calculated. Each sample was tested in triplicate and the data were presented as mean ± standard deviations. **b** Percentage inhibition of a panel of viruses was determined as described in the experimental procedures. The assay was performed in triplicate

To determine the antiviral effect of berberine, HIV-1 env-pseudotyped viruses were used to infect target cells. As shown in Fig. 6, berberine exhibited remarkable activities against HIV-1 IIIB, NL4-3 and Bal isolates with low IC50s in the microgram range of 6.356 ± 0.336 µg/ml, 5.574 ± 0.172 µg/ml and 5.586 ± 0.652 µg/ml, respectively. It also inhibited the infection of JRFL, JRCSF and AD8 with IC50s of 10.275 ± 1.215 µg/ml, 7.559 ± 0.646 µg/ml and 7.532 ± 0.454 µg/ml, respectively (Fig. 6a and Table 2). Berberine efficiently inhibited HIV-1 infection in a dose-dependent manner (Fig. 6a, b). It is important to determine the cytotoxic potential of berberine on the cell lines used in the antiviral assays. The cytotoxic concentration (CC50) of berberine was determined to be 107 μg/ml. Berberine showed low cytotoxicity.

**Table 2:** Berberine potently inhibited infection of HIV-1 Env-pseudotyped viruses

Virus	Clade	Tropism	IC50 (µg/ml)	
			T20	Berberine
IIIB	B	X4	0.193 ± 0.024	6.356 ± 0.336
NL4-3	B	X4	0.197 ± 0.035	5.574 ± 0.172
Bal	B	R5	0.18 ± 0.021	5.586 ± 0.652
JRFL	B	R5	0.58 ± 0.26	10.275 ± 1.215
JRCSF	B	R5	0.484 ± 0.131	7.559 ± 0.646
AD8	B	R5	0.607 ± 0.12	7.532 ± 0.454

## Discussion

HIV-1 entry into host cells is mediated by gp41 and represents an attractive target for therapeutic intervention. Previous studies showed that blocking HIV-1 entry into target cells plays a critical role in viral infections (Esté and Telenti 2007; Pierson and Doms 2003). X-ray crystallographic studies have confirmed that there are three grooves of the NHR trimer, each of the grooves has a highly conserved hydrophobic pocket that can be bound by both C-peptides and small-molecule entry inhibitors (Chan et al. 1998; Tan et al. 1997), such as D-peptides (Jiang et al. 1993; Wild et al. 1994), 5-Helix (Root et al. 2001) and IQN17-like molecules (Eckert and Kim 2001). The trimeric coiled-coil of gp41 serves as a potential target for designing anti-HIV peptides and small molecules.

Computer-aided molecular docking analysis suggested that berberine made several hydrophobic, polar interactions and hydrogen bonds with both the peptides N36 and C34 of gp41 to form a novel NHR-berberine-CHR complex before the formation of the 6-HB, to the best of our knowledge, this is the first report of identification of a new compound-peptide complex. Other studies demonstrated that the bisindole inhibitors showed the importance of the hydrophobic group to compound potency, it might interact with a tertiary component during membrane fusion (Zhou et al. 2019). NB-2 and NB-64 were selected from a drug-like chemical library and their acid groups interacted with residue Lys574 to form a salt bridge, and thus blocked the six-helix bundle formation and could inhibit HIV-1 infection (Jiang et al. 2004). The current model of HIV-1 Env membrane fusion predicted that CD4 binding resulted in the refolding of gp41 from a pre-hairpin intermediate to a hairpin conformation (Eggink et al. 2016; Jiang et al. 2015; Martinez Munoz et al. 2014). Env with mutations located outside the HR2 binding site affected fusion kinetics to decrease the sensitivity to VIR165. One explanation was that VIRIP analogs might target the FP during an intermediate (Eggink et al. 2019). In agreement with this model, binding of berberine to both heptad repeats of HIV-1gp41 in the prefusion state triggered conformational changes to form a random coil structure and distorted the natural 6HB conformation. NCCG-gp41 was designed by linking an N35 to the N-terminus of N34(L6)C28 polypeptide and could inhibit HIV-1 Env-mediated membrane fusion by targeting the gp41 CHR-helices in the fusion intermediate state (Louis et al. 2003). Jacobs et al. designed a covalent inhibitor specifically targeting residue Lys574, it could trap a pre-six-helix bundle fusion intermediate confirmation by a covalent reaction of Lys574 to facilitate covalent attachment (Jacobs et al. 2007), suggesting that interactions with both heptad repeats might be a general requirement for high-affinity binding of these small-molecule inhibitors of 6HB formation (Aneja et al. 2015; Rashad et al. 2015, 2017). The indole moiety of berberine pointed toward the mouth of the entrance cavity in the hydrophobic pocket of gp41 NHR and formed p–p interaction and amide-p stacked interaction. A hydrogen bond with T-639 and two polar bonds with Q-563 and T-639 were established, involving the oxygen and the C=O group of the ring, which appeared to make the most significant contribution to binding. Additional nine hydrophobic interactions with amino acids L-566, V-570, I-635, Y-638 and I-642 and other interactions with Q-563, Q-567, V-570, I-635, Y-638, and T-639 were also involved. The above analysis confirmed that berberine could interact with both the peptides N36 and C34 of gp41 and be able to disrupt the 6-HB formation, explaining the action mechanism of antiviral activities of berberine.

Our data found berberine inhibited syncytia formation in a dose-dependent manner with the IC50 value of 5.773 ± 0.457 μg/ml, it efficiently blocked fusion by causing a local disturbance of the natural 6HB conformation. Berberine completely inhibited six HIV-1 clade B isolates and exhibited remarkable activity against the IIIB, NL4-3, Bal, JRFL, JRCSF and AD8 isolates with IC50s in the microgram range of 6.356 ± 0.336 µg/ml, 5.574 ± 0.172 µg/ml, 5.586 ± 0.652 µg/ml, 10.275 ± 1.215 µg/ml, 7.559 ± 0.646 µg/ml and 7.532 ± 0.454 µg/ml, respectively, and hence suppressed the infectivity of HIV-1 in a concentration-dependent manner in all tested HIV-1 cell lines. The modified 23-residue C-peptides, bearing a sulfonic-γ-AA residue substitution and hydrocarbon stapling, could bind HIV-1 gp41 N-terminus and inhibit envelope-mediated membrane fusion in cell–cell fusion assays at nanomolar potency (Bolarinwa et al. 2018). These results suggest that berberine is a potent inhibitor against a broad spectrum of HIV-1 strains at low concentrations, in agreement with previous studies. Taken together, our results explore the possible binding mode of berberine and enhance our understanding of the action mechanism of HIV-1 entry into host cells and how viral fusion can be effectively inhibited.

It should be noted that this newly discovered complex could provide new clues for the molecular basis of the gp41-mediated membrane fusion. Berberine is proven safe and valid, non-toxic, cheap cost and wide-availability in resource and remains many pharmacological applications. And we successfully identify it as a novel and promising anti-HIV-1 inhibitor.

## Data Availability

The data and material during the current study are available from the corresponding author on reasonable request.

## References

[CR1] Aneja R, Rashad AA, Li H, Kalyana Sundaram RV, Duffy C, Bailey LD, Chaiken I (2015). Peptide triazole inactivators of HIV-1 utilize a conserved two-cavity binding site at the junction of the inner and outer domains of env gp120. J Med Chem.

[CR2] Asano S, Gavrilyuk J, Burton DR, Barbas CF (2014). 3rd Preparation and activities of macromolecule conjugates of the CCR5 antagonist maraviroc. ACS Med Chem Lett.

[CR3] Bikadi Z, Hazai E (2009). Application of the PM6 semi-empirical method to modeling proteins enhances docking accuracy of AutoDock. J Cheminform.

[CR4] Bolarinwa O, Zhang M, Mulry E, Lu M, Cai J (2018). Sulfono-γ-AA modified peptides that inhibit HIV-1 fusion. Org Biomol Chem.

[CR5] Chan DC, Kim PS (1998). HIV entry and its inhibition. Cell.

[CR6] Chan DC, Fass D, Berger JM, Kim PS (1997). Core structure of gp41 from the HIV envelope glycoprotein. Cell.

[CR7] Chan DC, Chutkowski CT, Kim PS (1998). Evidence that a prominent cavity in the coiled-coil of HIV type 1 gp41 is an attractive drug target. Proc Natl Acad Sci USA.

[CR8] Chun TW, Davey RT, Ostrowski M, Shawn Justement J, Engel D, Mullins JI, Fauci AS (2000). Relationship between pre-existing viral reservoirs and the re-emergence of plasma viremia after discontinuation of highly active anti-retroviral therapy. Nat Med.

[CR9] Darzynkiewicz Z, Zhao H, Halicka HD, Li J, Lee Y-S, Hsieh T-C, Wu J (2014). In search of anti-aging modalities: evaluation of mTOR- and ROS/DNA damage-signaling by cytometry. Cytom A.

[CR10] Dong H, Zhao Y, Zhao L, Lu F (2013). The effects of berberine on blood lipids: a systemic review and meta-analysis of randomized controlled trials. Planta Med.

[CR11] Dorr P, Westby M, Dobbs S, Griffin P, Irvine B, Macartney M, Mori J, Rickett G, SmithBurchnell C, Napier C, Webster R, Armour D, Price D, Stammen B, Wood A, Perros M (2005). Maraviroc (UK-427,857), a potent, orally bioavailable, and selective small-molecule inhibitor of chemokine receptor CCR5 with broad-spectrum anti-human immunodeficiency virus type 1 activity. Antimicrob Agents Chemother.

[CR12] Eckert DM, Kim PS (2001). Mechanisms of viral membrane fusion and its inhibition. Annu Rev Biochem.

[CR13] Eggink D, de Taeye SW, Bontjer I, Klasse PJ, Langedijk JP, Berkhout B, Sanders RW (2016). Hiv-1 escape from a peptidic anchor inhibitor through stabilization of the envelope glycoprotein spike. J Virol.

[CR14] Eggink D, Bontjer I, de Taeye SW, Langedijk JPM, Berkhout B, Sanders RW (2019). HIV-1 anchor inhibitors and membrane fusion inhibitors target distinct but overlapping steps in virus entry. J Biol Chem.

[CR15] Esté JA, Telenti A (2007). HIV entry inhibitors. Lancet.

[CR16] Halgren TA (1998). Merck molecular force field. I. Basis, form, scope, parameterization, and performance of MMFF94. J Comput Chem.

[CR17] Harmon B, Campbell N, Ratner L (2010). Role of Abl kinase and the Wave2 signaling complex in HIV-1 entry at a post-hemifusion step. PLoS Pathog.

[CR18] Hayashi K, Minoda K, Nagaoka Y, Hayashi T, Uesato S (2007). Antiviral activity of berberine and related compounds against human cytomegalovirus. Bioorg Med Chem Lett.

[CR19] Jacobs A, Quraishi O, Huang X, Bousquet-Gagnon N, Nault G, Francella N, Alvord WG, Pham N, Soucy C, Robitaille M, Bridon D, Blumenthal R (2007). A covalent inhibitor targeting an intermediate conformation of the fusogenic subunit of the HIV-1 envelope complex. J Biol Chem.

[CR20] Jiang SB, Lin K, Strick N, Neurath AR (1993). HIV-1 inhibition by a peptide. Nature.

[CR21] Jiang S, Lin K, Min L (1998). Conformation-specific monoclonal antibody reacting with fusion- active gp41 from the human immunodeficiency virus type 1 envelope glycoprotein. J Virol.

[CR22] Jiang S, Lu H, Liu S, Zhao Q, He Y, Debnath AK (2004). N substituted pyrrole derivatives as novel human immunodeficiency virus type 1 entry inhibitors that interfere with the gp41 six-helix bundle formation and block virus fusion. Antimicrob Agents Chemother.

[CR23] Jiang X, Feyertag F, Meehan CJ, McCormack GP, Travers SA, Craig C, Westby M, Lewis M, Robertson DL (2015). Characterizing the diverse mutational pathways associated with r5-tropic maraviroc resistance: Hiv-1 that uses the drug-bound CCR5 coreceptor. J Virol.

[CR24] Kilby JM, Hopkins S, Venetta TM, DiMassimo B, Cloud GA, Lee JY, Alldredge L, Hunter E, Lambert D, Bolognesi D, Matthews T, Johnson MR, Nowak MA, Shaw GM, Saag MS (1998). Potent suppression of HIV-1 replication in humans by T-20, a peptide inhibitor of gp41-mediated virus entry. Nat Med.

[CR25] Lieberman-Blum SS, Fung HB, Bandres JC (2008). Maraviroc: a CCR5-receptor antagonist for the treatment of HIV-1 infection. Clin Ther.

[CR26] Liu S, Lu H, Niu J, Xu Y, Wu S, Jiang S (2005). Different from the HIV fusion inhibitor C34, the anti-HIV drug Fuzeon (T-20) inhibits HIV-1 entry by targeting multiple sites in gp41 and gp120. J Biol Chem.

[CR27] Louis JM, Nesheiwat I, Chang L (2003). Covalent trimers of the internal N-terminal trimeric coiled-coil of gp41 and antibodies directed against them are potent inhibitors of HIV envelope-mediated cell fusion. J Biol Chem.

[CR28] Lu M, Blacklow SC, Kim PS (1995). A trimeric structural domain of the HIV-1 transmembrane glycoprotein. Nat Struct Biol.

[CR29] Martinez Munoz L, Barroso R, Dyrhaug SY, Navarro G, Lucas P, Soriano SF, Vega B, Costas C, Munoz-Fernandez MA, Santiago C, Frade JMR, Franco R, Mellado M (2014). CCR5/CD4/CXCR4 oligomerization prevents HIV-1 gp120iiib binding to the cell surface. Proc Natl Acad Sci USA.

[CR30] Miyauchi K, Kozlov MM, Melikyan GB (2009). Early steps of HIV-1 fusion define the sensitivity to inhibitory peptides that block 6-helix bundle formation. PLoS Pathog.

[CR31] Mohan MC, Abhimannue AP (2017). Identification and characterization of berberine in *Tinospora cordifolia* by liquid chromatography quadrupole time of flight mass spectrometry (LC-MS/MS Q-tof) and evaluation of its anti inflammatory potential. Pharmacogn J.

[CR32] Morris GM, Goodsell DS, Halliday RS, Huey R, Hart WE, Belew RK, Olson AJ (1998). Automated docking using a Lamarckian genetic algorithm and an empirical binding free energy function. J Comput Chem.

[CR33] Munoz-Barroso I, Durell S, Sakaguchi K, Appella E, Blumenthal R (1998). Dilation of the human immunodeficiency virus-1 envelope glycoprotein fusion pore revealed by the inhibitory action of a synthetic peptide from gp41. J Cell Biol.

[CR34] Pierson TC, Doms RW (2003). HIV-1 entry and its inhibition. Curr Top Microbiol Immunol.

[CR35] Pleskoff O, Treboute C, Brelot A, Heveker N, Seman M, Alizon M (1997). Identification of a chemokine receptor encoded by human cytomegalovirus as a cofactor for HIV-1 entry. Science.

[CR36] Rashad AA, Kalyana Sundaram RV, Aneja R, Duffy C, Chaiken I (2015). Macrocyclic envelope glycoprotein antagonists that irreversibly inactivate HIV-1 before host cell encounter. J Med Chem.

[CR37] Rashad AA, Acharya K, Haftl A, Aneja R, Dick A, Holmes AP, Chaiken I (2017). Chemical optimization of macrocyclic HIV-1 inactivators for improving potency and increasing the structural diversity at the triazole ring. Org Biomol Chem.

[CR38] Refaat A, Abdelhamid S, Saiki I, Sakurai H (2015). Inhibition of p38 mitogen-activated protein kinase potentiates the apoptotic effect of berberine/tumor necrosis factor-related apoptosis-inducing ligand combination therapy. Oncol Lett.

[CR39] Roche S, Albertini AA, Lepault J, Bressanelli S, Gaudin Y (2008). Structures of vesicular stomatitis virus glycoprotein: membrane fusion revisited. Cell Mol Life Sci.

[CR40] Root MJ, Kay MS, Kim PS (2001). Protein design of an HIV-1 entry inhibitor. Science.

[CR41] Solis FJ, Wets RJB (1981). Minimization by random search techniques. Math Oper Res.

[CR42] Stermitz FR, Lorenz P, Tawara JN, Zenewicz LA, Lewis K (2000). Synergy in a medicinal plant: antimicrobial action of berberine potentiated by 5′-methoxyhydnocarpin, a multidrug pump inhibitor. Proc Natl Acad Sci USA.

[CR43] Tan K, Liu J, Wang S, Sgen S, Lu M (1997). Atomic structure of a thermostable subdomain of gp41. Proc Natl Acad Sci USA.

[CR44] Varghese FS, Thaa B, Amrun SN, Simarmata D, Rausalu K, Nyman TA, Merits A, McInerney GM, Ng LFP, Ahola T (2016). The antiviral alkaloid berberine reduces chikungunya virus-induced mitogen-activated protein kinase signaling. J Virol.

[CR45] Wang HQ, Li K, Ma LL, Wu S, Hu J, Yan HY, Jiang JD, Li YH (2017). Berberine inhibits enterovirus 71 replication by downregulating the MEK/ERK signaling pathway and autophagy. Virol J.

[CR46] Weissenhorn W, Wharton SA, Calder LJ, Earl PL, Moss B, Aliprandis E, Skehel JJ, Wiley DC (1996). The ectodomain of HIV-1 env subunit gp41 forms a soluble, alpha-helical, rod-like oligomer in the absence of gp120 and the N-terminal fusion peptide. EMBO.

[CR47] Weissenhorn W, Dessen A, Harrison SC, Skehel JJ, Wiley DC (1997). Atomic structure of the ectodomain from HIV-1 gp41. Nature.

[CR48] Wild CT, Shugars DC, Greenwell TK, Mc-Danal CB, Matthews TJ (1994). Peptides corresponding to a predictive alpha-helical domain of human immunodeficiency virus type 1 gp41 are potent inhibitors of virus infection. Proc Natl Acad Sci.

[CR49] Wu Y, Li JQ, Kim YJ, Wu J, Wang Q, Hao Y (2011). In vivo and in vitro antiviral effects of berberine on influenza virus. Chin J Integr Med.

[CR50] Yan YQ, Fu YJ, Wu S, Qin HQ, Zhen X, Song BM, Weng YS, Wang PC, Chen XY, Jiang ZY (2018). Anti-influenza activity of berberine improves prognosis by reducing viral replication in mice. Phytother Res.

[CR51] Zhou G, Chu S, Nemati A, Huang C, Snyder BA, Ptak RG, Gochin M (2019). Investigation of the molecular characteristics of bisindole inhibitors as HIV-1 glycoprotein-41 fusion inhibitors. Eur J Med Chem.

